# Deep learning-based debris flow hazard detection and recognition system: a case study

**DOI:** 10.1038/s41598-025-86471-4

**Published:** 2025-02-25

**Authors:** Fei Wu, Jianlin Zhang, Dunlong Liu, Andreas Maier, Vincent Christlein

**Affiliations:** 1 School of Electrical, Electronics and Communication Engineering, University of Chinese Academy of Sciences, Beijing, 100049 China; 2 National Key Laboratory of Optical Field Manipulation Science and Technology, Key Laboratory of Optical Engineering, Institute of Optics and Electronics, Chinese Academy of Sciences, Chengdu, 610209 China; 3 Pattern Recognition Lab, Department of Computer Science, Friedrich-Alexander-Universität Erlangen-Nürnberg, 91058 Erlangen, Germany; 4 Software Automatic Generation and Intelligent Service Key Laboratory of Sichuan Province, Chengdu University of Information and Technology, Chengdu, 610225 China

**Keywords:** Transfer learning, Convolutional neural network, Debris flow, Hazard detection and recognition, Natural hazards, Computer science

## Abstract

Debris flows are characterized by their suddenness, rapidity, large scale and destructive power, causing serious threat to the population in mountainous areas. Surveillance cameras are widely used in geological hazard monitoring and early warning projects. So far, video cameras are used as a passive tool for post inspection and not as an active role for debris flow monitoring and early warning. Inspired by recent developments of anomaly detection in the field of computer vision, in this paper, we propose a novel automatic debris flow detection and recognition system based on deep learning. It consists of a video feature extraction network using a 3D convolutional neural network (CNN), a debris flow hazard detection network using a multi-layer perceptron (MLP), and a debris flow hazard recognition network for verification employing another CNN. The proposed system takes the video sequences captured by the cameras as inputs and enables the detection and recognition of debris flow hazards. All the networks are optimized and evaluated on a newly annotated image dataset called Debrisflow23. Extensive experimental evaluations with a detection accuracy of 86.3 % AUC, a recognition accuracy of 83.7 % AUC, and an overall identification accuracy of 88.1 % AUC on the test dataset demonstrate that the proposed method possesses accurate and reliable debris flow warning capability. Thus, further precautions can be taken in advance to reduce the damage to human settlements and infrastructure caused by debris flows.

## Introduction

Debris flows are common and destructive mountain disasters, which contain a fluid mixture of loose muds, silts, soils, rocks, and water that flow downstream at high speed^1–3^. They occur suddenly and are difficult to ward off, so that people in the mountain regions have suffered great loss of life and property^4–6^. The extent and frequency of debris flows have increased in recent years, causing devastating damage in the affected areas^6^. The scale of debris flow disasters in China is large^7^, making it impossible to provide comprehensive engineering treatment. Therefore, as an important non-engineer measure for disaster reduction and an effective measure for debris flow disaster prevention, debris flow monitoring and early warning technology is highly valued by academic and engineering domains^8,9^.

Most of the current debris flow monitoring and early warning technologies are based on natural indicators, such as rainfall, soil moisture content, infrasound, earth sound or mud water level^5,7,10–13^. The movement of debris flow masses can produce corresponding ground vibration and infrasound waves and are thus a major technique in debris flow monitoring process^14–21^. Zhang and Yu^22,23^ developed a debris flow infrasound monitoring equipment for debris flow observation in Jiangjia gully, China. It can be used as an early warning system for debris flows by integrating rain gauges, image transmission and other functions. Kogelnig et al.^24,25^ compared and verified the infrasound signal of debris flows from different flow depth and corresponding ground sound. They pointed out that the joint application of infrasound and ground sound has great potential for monitoring debris flows. Li et al.^6^ developed a debris flow infrasound monitoring device to observe the dynamic changes of debris flows along a railroad line in China. Later, Liu et al.^26^ analyzed the characteristic difference between debris flow infrasound and environmental interference infrasound, and proposed a recognition system for debris flow infrasound by adjusting the threshold of characteristic parameters. Liu et al.^27^ established a sound source locative model based on an infrasound monitoring array and a cross-correlation time delay estimation algorithm. They realized a real-time debris flow movement localization and tracking based on a GIS platform. Based on the KNN algorithm, Liu et al.^28^ proposed an automatic signal recognition model for analyzing the key characteristics of landslide infrasound and various common environmental interference infrasound from the time and frequency domains, respectively. Conversely, Zhang et al.^29^ proposed a debris flow I-D threshold curve for plotting the relationship between rainfall parameters and debris flow density based on a physical model of debris flow formation mechanism and fluid properties. It can provide real-time debris flow warnings based on the monitored rainfall data. Kai-heng and Chao^30^ established a debris flow early warning method by constructing the empirical relationship among the critical soil moisture content, the soil permeability coefficient, and the porosity and particle curvature coefficients. Concurrently, Zhao et al.^11^ utilized rainfall gauge, mud level gauge, infrasound alarm instrument and video monitoring equipment to formulate warning thresholds and determine warning levels. Xie et al.^31^ selected daily maximum temperature, daily rainfall, mud water level, surface displacement, and water content as debris flow monitoring and early warning indicators. By hierarchically weighting these indicators, they proposed a glacier rainfall debris flow warning model based on the excitation conditions and the stability of the accumulation body.

However, sensor alarm systems comprise a complex set of equipment that is costly to install, operate and maintain, resulting in a lack of widespread application in many undeveloped areas of the world^3^. In contrast, an increasing number of video cameras are being installed along the torrents that are prone to debris flows. However, they can only be used as a passive tool for retrospective inspection to capture, verify, and record the course of disasters, losing the role of real-time monitoring and early warning. Therefore, effective utilization of debris flow events captured by video cameras can provide valuable information for debris flow detection and monitoring. Several works used video cameras for assessing the movement of debris flows. Uddin et al.^32–34^ developed computer-based spatial-filtering velocimeters to measure the surface velocity of natural debris flows from Mt. Yakedake Volcano while Arattano and Marchi^35^ proposed an image processing technique to calculate velocity distribution of a debris flow event in Moscardo. Nevertheless, these methods only analyzed the debris flow velocity distribution, and thus cannot achieve recognition and early warning from the appearance of debris flow images acquired by the video cameras.

In recent years, with the development of computer vision technology, various deep learning methods based on digital images have been proposed for applications in the field of disaster response. Rahnemoonfar et al.^36^ proposed an end to end densely connected convolutional neural network (CNN) and recurrent neural network (RNN) model for detecting flooded areas in unmanned aerial vehicles (UAV) aerial images. Based on transfer learning, Pi et al.^37^ evaluated eight You-Only-Look-Once (YOLO) models^38^ for detecting post-disaster ground objects from aerial views. Similarly, Pham and Kim^3^ used an advanced YOLO network YOLOv4^39^ for debris flow detection and identification while Ji et al.^40^ equipped a residual neural network (ResNet-50) model^41^ with a novel attention module for landslides detection from satellite images. So far, these existing CNN-based methods have succeeded in accurately detecting objects from the images of cameras, UAVs, and drones. However, to the best of our knowledge, there is no previous research reported on using continuous surveillance video sequences for monitoring and early warning of debris flow hazards. As discussed above, Pham and Kim^3^ used object detection model YOLOv4^39^ to directly detect debris flow hazards with rectangular bounding boxes on the debris flow images, while our goal is to predict the debris flow motion tendency based on inter-frame context information of consecutive video frames, and detail the extent of debris flow hazards.

On the other hand, anomaly detection is one of the most challenging and perennial problems in computer vision, e. g.,  detecting violence or aggression in video sequences^42–48^. Datta et al.^49^ proposed to detect human violence by exploiting the trend of human movement and limb direction. Kooij et al.^50^ analyzed video and audio data to detect attacks in surveillance videos while Gao et al.^51^ proposed violent flow descriptors to detect human violence. At the same time, Mohammadi et al.^52^ proposed a new classification method to identify violent or nonviolent videos based on behavioral heuristics. More recently, Sultani et al.^53^ proposed an anomaly detection framework to detect video segment level anomaly based on deep learning, which arouses lots of attention from researchers^54–57^.

The aim of this work is to propose a new method for detecting debris flow hazards and to further verify the occurrence of debris flows based on the video sequence inputs recorded by cameras. We built a unified debris flow detection and recognition system based on a combination of a video feature extraction network, a debris flow hazard detection network, and a debris flow hazard recognition network. The first step in debris flow hazard detection is the conversion of the captured video clips into corresponding anomaly scores. Then, an additional hazard identification process is designed to scrutinize the results of the debris flow detection based on pre-determined hazard thresholds. As a consequence, the proposed system provides accurate and reliable identification and early warning for debris flow hazards. Additionally, realistic natural videos acquired from fixed cameras at different sampling sites in China were collected and annotated to optimize and evaluate the deep learning models within the proposed system. Combined with state-of-the-art computer vision techniques, we believe that this study can provide new inspirations for debris flow detection and identification in early warning and monitoring systems.

The rest of the paper is organized as follows: “Methodology” demonstrates the details of the proposed method by elaborating the datasets, network architectures, and component structures. “Experiments” shows the experimental setups and the evaluation results for the proposed method, while its limitations and future works are discussed in “Discussion”. Finally, we conclude the article in “Conclusion”.

**Fig. 1:**
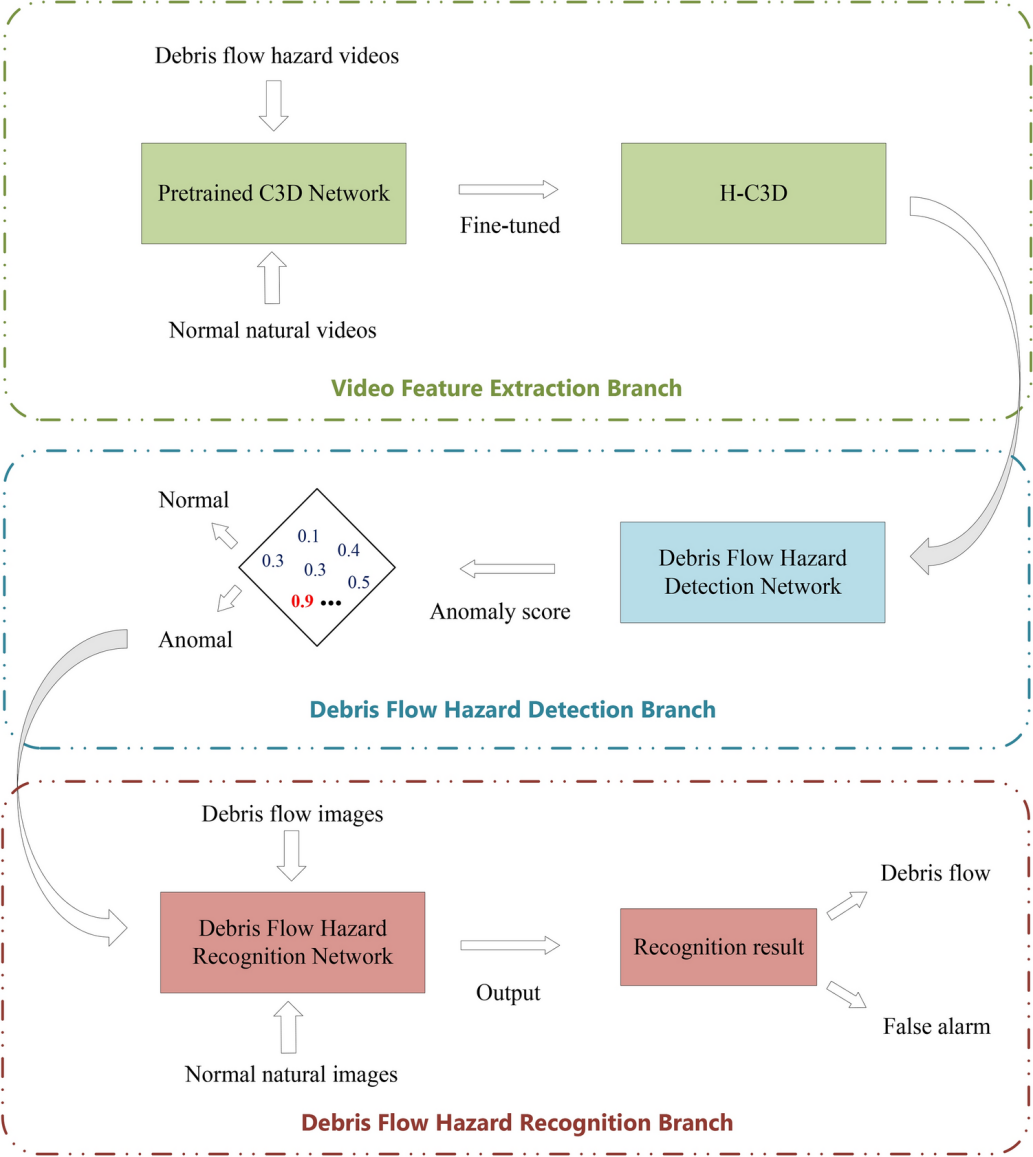
The overall methodology of the proposed method. It consists of a video feature extraction branch, a debris flow hazard detection branch and a debris flow hazard recognition branch.

## Methodology

In this section, we present the deep learning-based debris flow detection and recognition system. The collected data used in this work are presented in “Dataset”. The overall methodology of this work can be seen in Fig. 1. The system consists of a video feature extraction branch (“Transfer learning”), a debris flow hazard detection branch (“Debris flow hazard detection network”) and a debris flow hazard recognition branch (“Debris flow hazard recognition network”). The network architecture and hyper-parameter settings of each subbranch are detailed in the corresponding subsections.

**Fig. 2:**
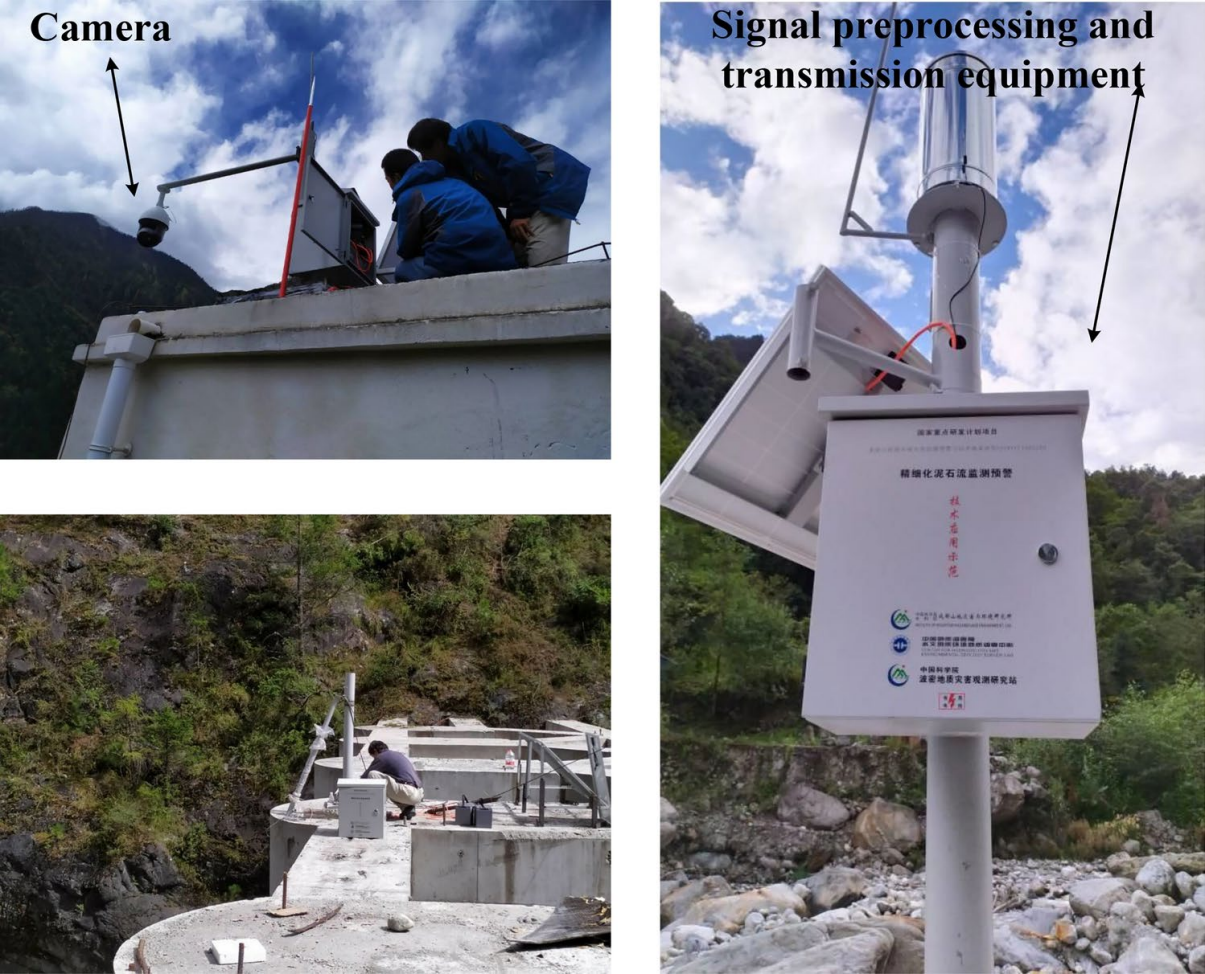
Cameras and signal preprocessing and transmission equipment were installed to monitor debris flow hazards at the sampling sites in China.

**Fig. 3:**
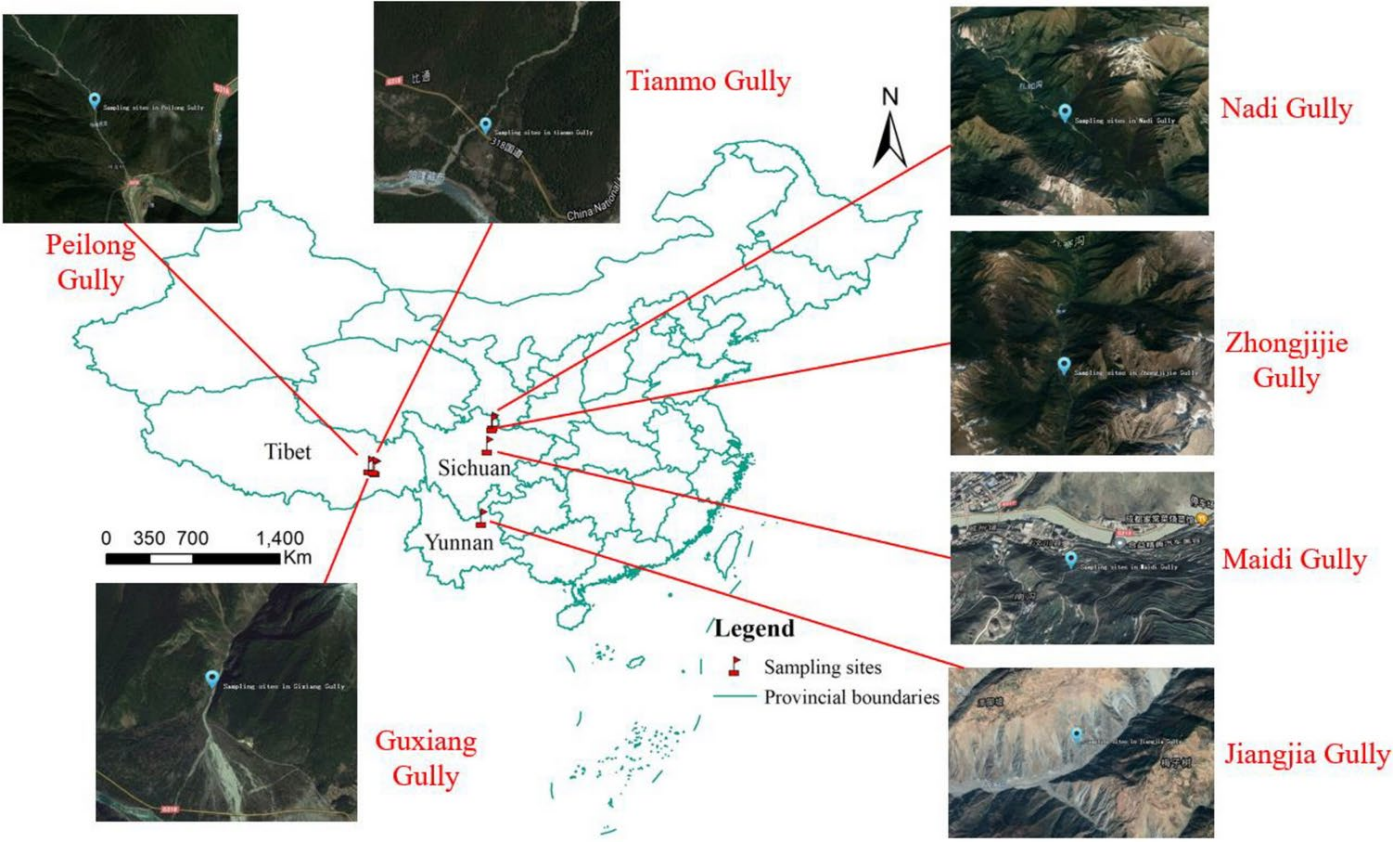
The 7 sampling sites for monitoring debris flow hazards in China.

### Dataset

To facilitate the understanding of the subsequent experiments, we first give a summary of the collected dataset used in this work. As shown in Fig. 2, cameras with signal preprocessing and transmission equipment were installed at 7 sampling sites in China that are vulnerable to debris flow hazards (Peilong Gully, Tianmo Gully and Guxiang Gully of Tibet, Jiangjia Gully of Yunnan, and Nadi Gully, Zhongjijie Gully and Maidi Gully of Sichuan). Figure 3 illustrates the locations of these sites. We collected 12 debris flow hazard videos and 12 normal natural videos from these sampling sites. To further extend the available data, we searched and collected 11 additional debris flow hazard videos and 11 normal natural videos from the Internet. All these collected videos were mixed and then annotated with image-level labels: debris flow vs. normal natural. Except for the debris flow images in debris flow hazard videos, the rest are normal natural images. The resulting image dataset is named Debrisflow23. With a random selection scheme, we split the Debrisflow23 dataset into a training set (12 debris flow hazard videos and 12 normal natural videos), a validation set (4 debris flow hazard videos and 4 normal natural videos), and a test set (7 debris flow hazard videos and 7 normal natural videos). Table 1 summarizes the detailed properties of the Debrisflow23 dataset used in this work.

**Table 1:** Debrisflow23 dataset statistics.

	Video label	Number	Total frames	Debris flow frames
Training	Debris flow	12	18960	13697
	Normal natural	12	21552	0
Validation	Debris flow	4	9072	4437
	Normal natural	4	7648	0
Test	Debris flow	7	13837	8993
	Normal natural	7	12431	0
Total	Debris flow	23	41869	27127
	Normal natural	23	41631	0

**Fig. 4:**
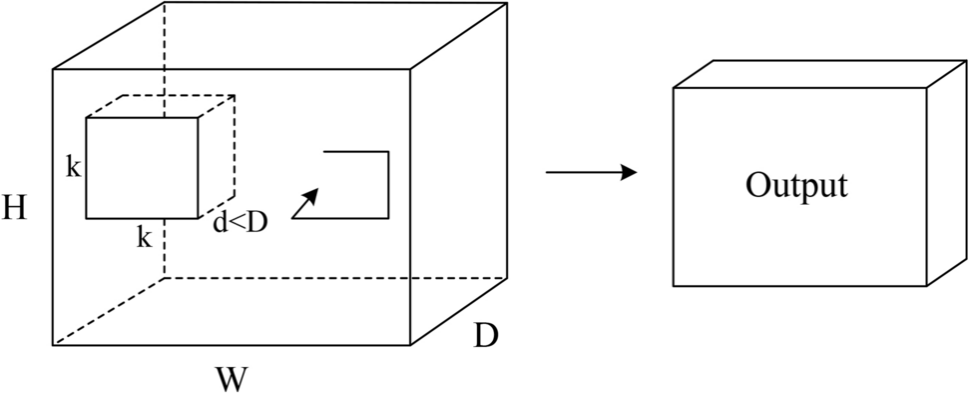
A 3D convolutional operation^58^, H, W, D denotes the height, width and depth of the video data cube, d denotes kernel temporal depth and k denotes kernel spatial size.

**Fig. 5:**
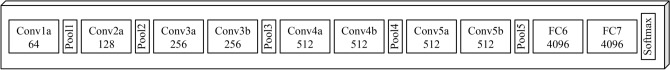
The C3D network architecture^58^ used for transfer learning.

### Transfer learning

As the first step towards monitoring and early warning of debris flow hazards from 3D surveillance videos, a video feature extraction network is required. The C3D network^58^ was a representative method proposed for video feature learning. It uses a 3D convolutional neural network for feature extraction on video sequence images. A 3D convolutional operation^58^, as shown in Fig. 4, is a key design for extracting 3D feature of video sequences. That means multiple continuous images of the sequence in the video are stacked to form a cube, while the 3D convolutional kernel is used to perform sliding window convolution in the height, width and depth direction of this cube to generate 3D feature maps. In contrast to standard 2D convolution^59^, the kernel depth of 3D convolution is smaller than the number of cube channels ($\mathrm {d<D}$), thus the 3D convolutional kernel can convolve the video cube in the depth direction. As a result, the motion information along the time direction of the stacked images cube can be captured, resulting in various successful applications in computer vision tasks, such as action recognition^60,61^, 3D medical segmentation^62,63^, etc.

To adapt C3D^58^ to our debris flow hazard detection task, transfer learning is applied to the original C3D network^58^. The architecture of C3D network is illustrated in Fig. 5. We follow the original hyperparameter settings of C3D to fine-tune the pretrained C3D model with the video-level-label-based training set of Debrisflow23 (“Dataset”), the resulting model, named H-C3D, is used to extract 3D features of video sequences used in this work.

**Fig. 6:**
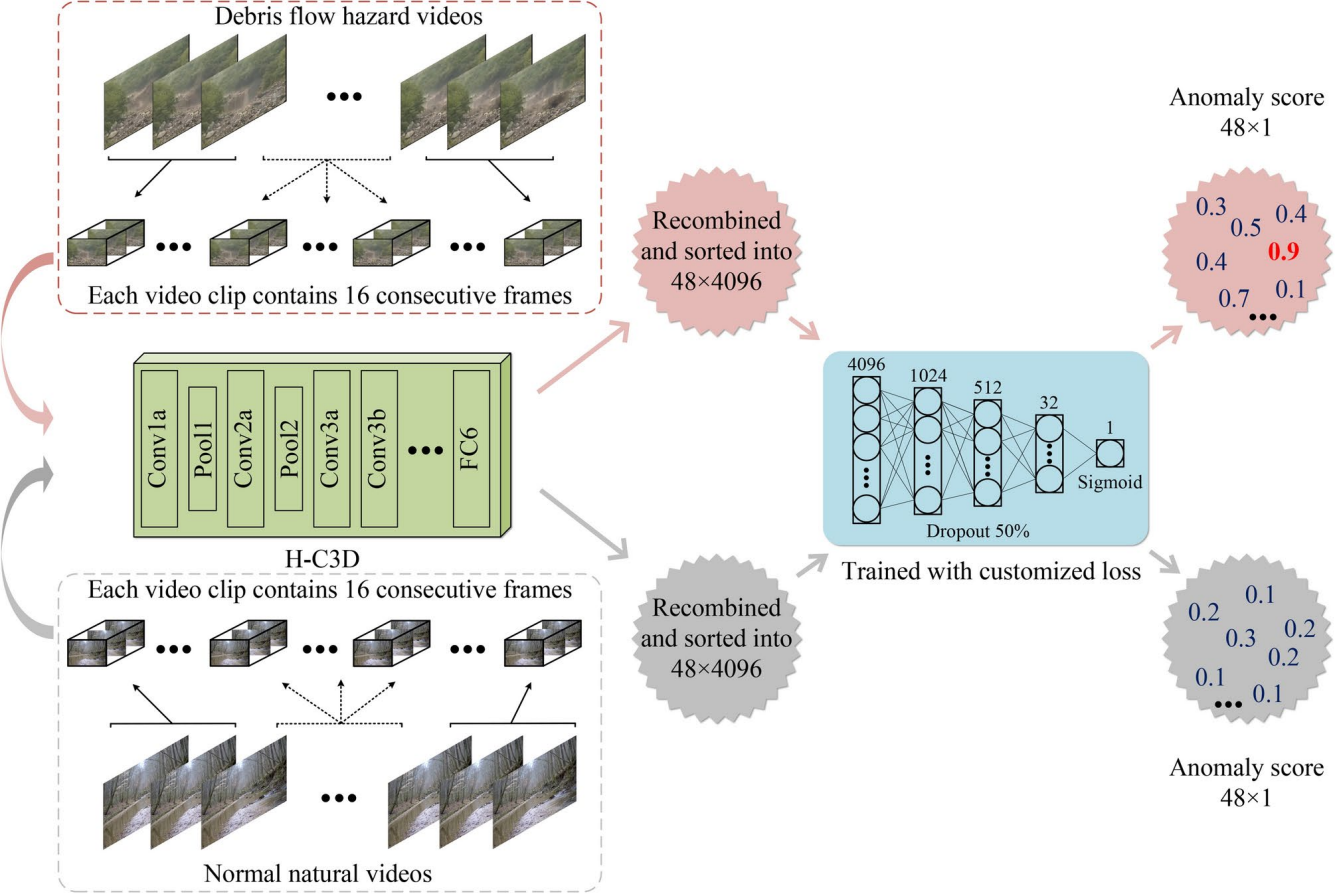
Overview of the combination of the trimmed H-C3D (green box) and the debris flow hazard detection network (blue box). The debris flow hazard detection network takes the 3D features of the video clips generated by H-C3D as input and outputs the corresponding 0 to 1 anomaly score.

### Debris flow hazard detection network

Inspired by the recent developments of abnormal behavior detection in computer vision^53–58^, we design a debris flow hazard detection network to detect the debris flow hazard from the obtained 3D features of H-C3D in consecutive video frames. The aim of the debris flow hazard detection network is to use the 3D features generated by H-C3D of corresponding video clips as input and output anomaly scores w. r. t. the 3D features, thus enabling an initial detection of debris flow hazards.

The overview of the combination of the trimmed H-C3D and the debris flow hazard detection network is shown in Fig. 6. In particular, H-C3D takes a 16-frame video clip as input and outputs the corresponding 3D feature from the first fully connected layer (FC6). The FC6 can generate a 4096-dimensional feature vector which represents high-level semantic information. Each video in the training dataset is sequentially divided into 16 consecutive image frames to produce an $N\times 4096$ dimensional feature vectors through the H-C3D network, where N denotes the maximum value of the total number of corresponding video frames divided by 16. Afterwards, these feature vectors are recombined and sorted into a $48\times 4096$ dimensional feature vector in a chronological order. The debris flow hazard detection network adopts a five-layer fully connected neural network aiming to transform the 4096-dimensional 3D vector into a 1-dimensional 0 to 1 anomaly score which is obtained by a Sigmoid activation function. All other layers employ ReLU activation and 50 % dropout for non-linearity and regularization, respectively. In this way, each 3D feature generates an anomaly score ranging from 0 to 1, resulting in a total of $48\times 1$ dimensional score sequences corresponding to the input video.

To smooth the anomaly response curve while reducing false alarms in the early warning of debris flow disaster, we specifically designed a loss function based on multiple instance learning (MIL) for optimizing the debris flow hazard detection network. The main idea of this design is as follows:

1 $$\begin{aligned} \mathop {\textrm{max}}\limits _{{i\in {B_{d}}}}\varphi {(V_{d}^{i})}-\mathop {\textrm{min}}\limits _{i\in {B_{d}}}\varphi {(V_{d}^{i})}>\mathop {\textrm{max}}\limits _{j\in {B_{n}}}\varphi {(V_{n}^{j})}-\mathop {\textrm{min}}\limits _{j\in {B_{n}}}\varphi {(V_{n}^{j})} \end{aligned}$$

where $\varphi (*)$ represents the debris flow hazard detection network embedding. $B_{d}$ and $B_{n}$ represent the generated video clips (instances) from all the debris flow hazard videos and all the normal natural videos, respectively. $V_{d}^{i}$ and $V_{n}^{j}$ represent the $i^\text {th}$ and $j^\text {th}$ video sequence from debris flow hazard videos and normal natural videos, respectively. Equation (1) aims to make the difference between the maximum and the minimum anomaly score of debris flow hazard videos larger than the difference between the maximum and the minimum score of normal natural videos. This can be formulated into the following loss function:

2 $$\begin{aligned} {\mathscr {L}}_1 = \textrm{max}\{0, 1-\big (\mathop {\textrm{max}}\limits _{i\in {B_{d}}}\varphi {(V_{d}^{i})}-\mathop {\textrm{min}}\limits _{i\in {B_{d}}}\varphi {(V_{d}^{i})} -(\mathop {\textrm{max}}\limits _{j\in {B_{n}}}\varphi {(V_{n}^{j})}-\mathop {\textrm{min}}\limits _{j\in {B_{n}}}\varphi {(V_{n}^{j})})\big )\} . \end{aligned}$$

Similar to previous anomaly detection works^53,56,57^, temporal smoothness and sparsity smoothness constraints are added to form the final loss function. The temporal smoothness constraint aims to minimize the response differences between the temporally adjacent debris flow hazard video segments:

3 $$\begin{aligned} {\mathscr {L}}_2 = \sum _{k=1}^{47}(\varphi {(V_{d}^{i^{k}})-\varphi {(V_{d}^{i^{k+1}})}})^2 , \end{aligned}$$

where $\varphi {(V_{d}^{i^{k}})}$ indicates the $k^\text {th}$ instance score of $i^\text {th}$ video sequence from debris flow hazard videos. The Sparsity smoothness constraint aims to minimize the response of each debris flow hazard video segments as follows:

4 $$\begin{aligned} {\mathscr {L}}_3 = \sum _{k=1}^{48}\varphi {(V_{d}^{i^{k}})} . \end{aligned}$$

Eventually, the objective loss function of the debris flow hazard detection network can be summarized as:

5 $$\begin{aligned} {\mathscr {L}}_{\text {final}} = {\mathscr {L}}_1 + \lambda _t \,{\mathscr {L}}_2 + \lambda _s \, {\mathscr {L}}_3 , \end{aligned}$$

where $\lambda _t,\lambda _s$ are trade-off hyper-parameters that represent temporal (*t*) and spatial (*s*) smoothness constraints, respectively. In this way, the loss function makes the maximum anomaly score of debris flow hazard video segments to be 1, while suppressing the maximum anomaly score of normal natural video segments to be 0.

**Fig. 7:**
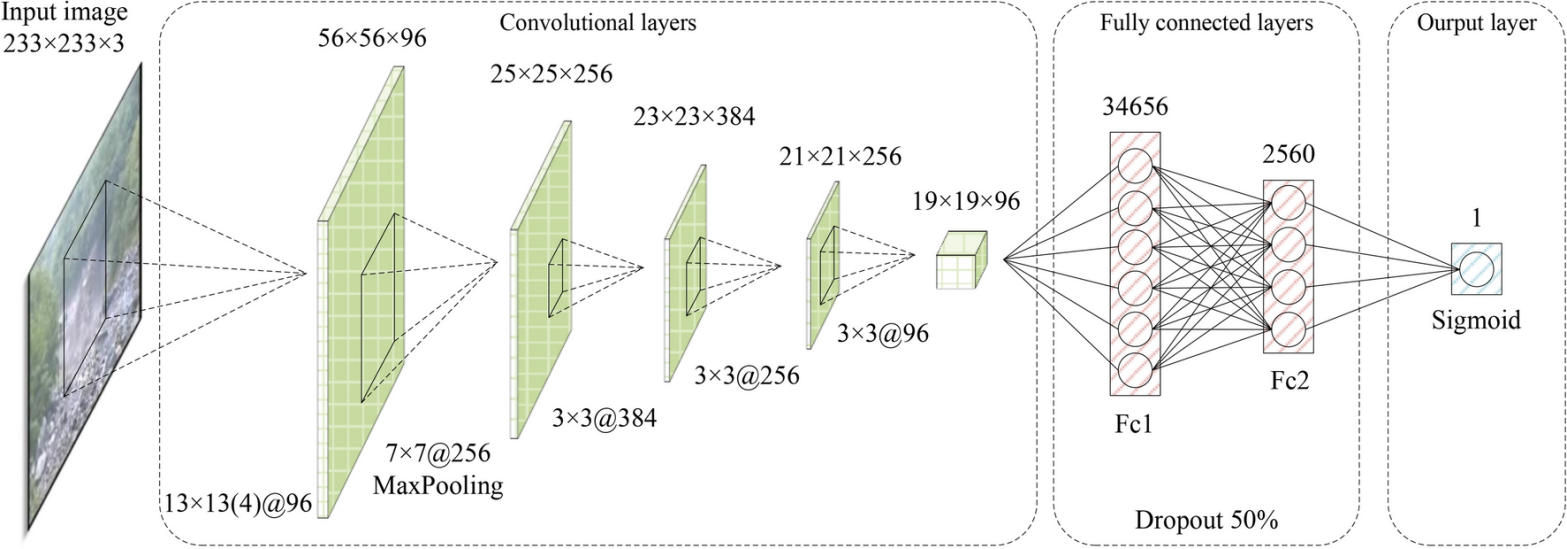
Architectural details of the debris flow hazard recognition network.

### Debris flow hazard recognition network

The combination of the H-C3D and the debris flow hazard detection network predicts the debris flow motion tendency based on inter-frame context information of consecutive video frames from a video-level perspective. To further check the potential false alarms associated with high anomaly scores in some noise cases, the initial debris flow detection results generated from the debris flow hazard detection network are re-evaluated. In particular, after obtaining the initial debris flow detection results, a debris flow hazard recognition network is designed to verify the occurrence of a debris flow hazard disaster from an image-level perspective, thereby reducing the false alarm rate. The architectural details of the debris flow hazard recognition network is shown in Fig. 7. It adopts five convolutional layers, two fully connected layers and a final linear layer with Sigmoid activation. The image-level samples with a size of 233 $\times$ 233 pixels from the debris flow hazard videos and normal natural videos of the Debrisflow23 training set (Section 2.1) are used as input for the debris flow hazard recognition network. While the binary cross entropy (BCE) loss is used for network optimization. During inference, we first set up a threshold (empirically set to $\theta =0.6$) based on the anomaly score $\delta \in [0,1]$ generated by the debris flow hazard detection network to identify the initial detection of a debris flow hazard. The verification process is then conducted on this initial debris flow hazard detection result, which can be represented as follows:

6 $$\begin{aligned} \text {Recognition result}= {\left\{ \begin{array}{ll} \text {Abnormal}\quad \delta>\theta \quad {\left\{ \begin{array}{ll} \text {Debris flow}\quad & \phi (I)>0.5 \\ \text {False alarm}\quad & \text {otherwise} \end{array}\right. } \\ \text {Normal}\quad \text {otherwise} \end{array}\right. } \end{aligned}$$

where $\phi (I)$ represents the debris flow hazard recognition network embedding $\phi (*)$ for the image-level input *I*. The verification process makes the final identification of the proposed system to be either a debris flow hazard or a normal case (normal event and false alarm).

**Table 2:** Model optimization settings for the proposed method.

Network	Optimizer	Loss function	Batch size	Iteration	Initial learning rate
# 1	SGD	Softmax loss	50	20000	0.001
# 2	Adagrad	Equation (5)	24	20000	0.01
# 3	Adagrad	BCE loss	128	8000	0.01

## Experiments

In this section, we present the implementation details of our experimental setup as well as analyzing the experimental results in both quantitative and qualitative perspectives.

### Implementation details

The proposed debris flow hazard detection and recognition system is implemented on a PC equipped with an Intel Core i7-9700k@3.6 GHz CPU and a single Nvidia GeForce RTX 2070SUPER GPU. The user interface is developed using Python 3.7 PyQt5. The hyperparameter settings for model optimization of the video feature extraction network (# 1), debris flow hazard detection network (# 2), and debris flow hazard recognition network (# 3) are listed in Table 2. The $\lambda _t,\lambda _s$ in Eq. (5) are both set to 8e-5. During training, the validation set of Debrisflow23 (Section 2.1) is used to validate the model performance and define the best model.

**Table 3:** Confusion matrix.

		Prediction	
		Predicted positive	Predicted negative
Ground truth	Positive	True positives (TP)	False negatives (FN)
	Negative	False positives (FP)	True negatives (TN)

**Fig. 8:**
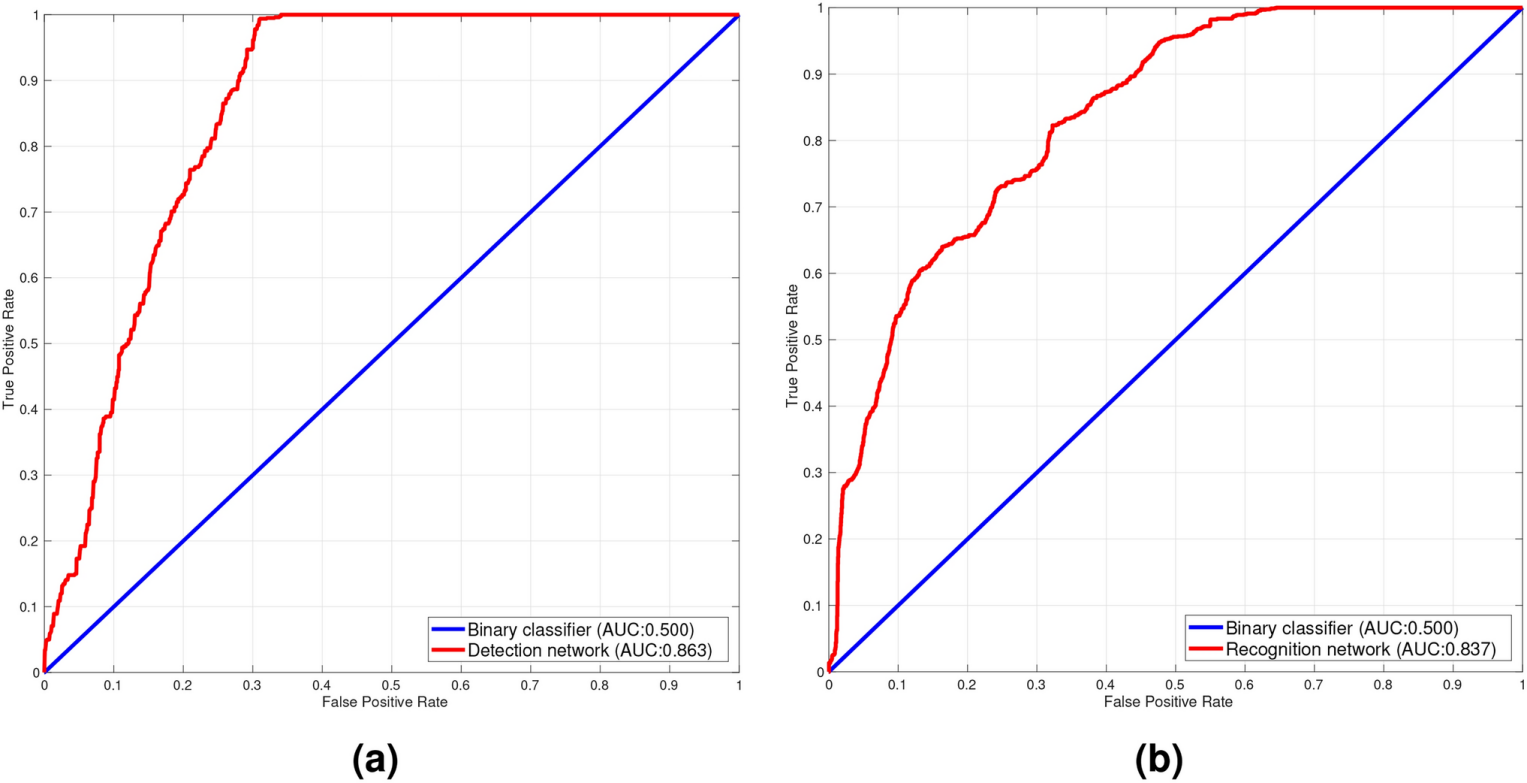
ROC curves of the debris flow hazard detection network (**a**) and debris flow hazard recognition network (**b**).

**Table 4:** Effectiveness and efficiency evaluation for the proposed method.

Network	Model size	Speed (FPS)	Accuracy
# 1	312.0M	113	N/A
# 2	18.9M	849	0.863
# 3	367.9M	415	0.837
# 4	698.8M	68	0.881

**Fig. 9:**
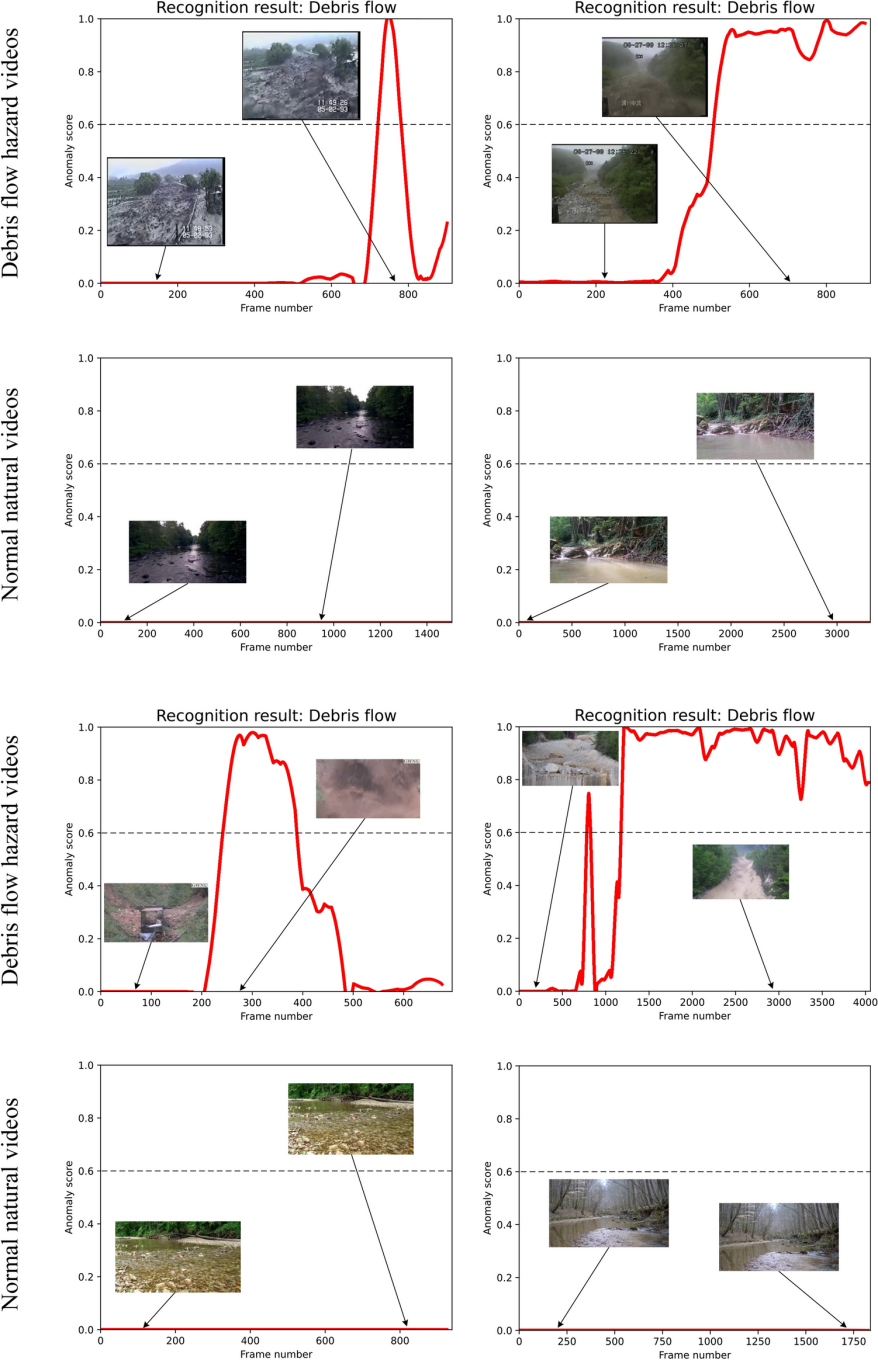
Qualitative results of the proposed method on several test videos (best viewed zoomed in).

**Fig. 10:**
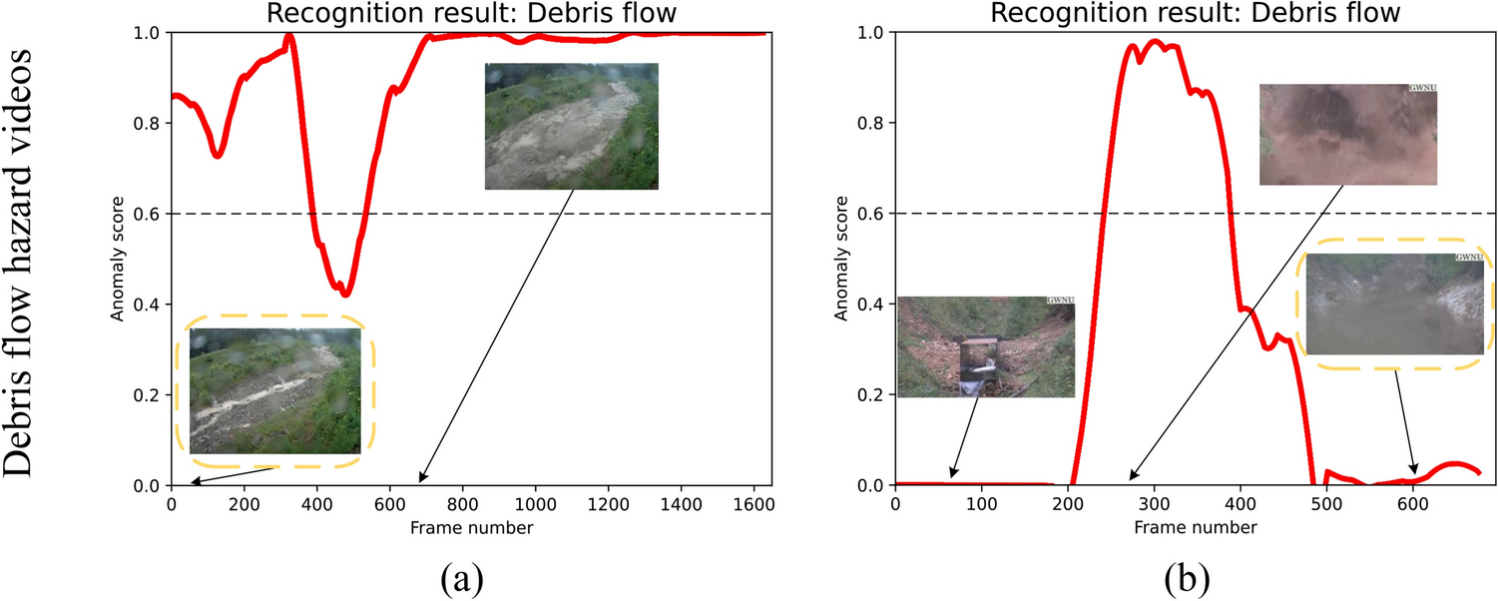
Failure cases of the debris flow hazard detection network with false alarms (**a**) and wrong detections (**b**). The yellow dotted boxes indicate that relying solely on the detection network is unable to identify the debris flow hazard correctly (best viewed zoomed in).

### Quantitative results

To consistently evaluate the debris flow detection and recognition performance from the debris flow hazard detection network and debris flow hazard recognition network, a frame-based receiver operating characteristics (ROC) curve is used as evaluation criterion. The ROC curve is a widely applied standard for visualizing the trade-offs between sensitivity and specificity in a binary classifier with the corresponding area under the curve (AUC) including all the possible decision thresholds from a diagnostic test result. The ROC criterion consists of true positive rate (TPR) and false positive rate (FPR). TPR and FPR are defined as follows:

7 $$\begin{aligned} \text {TPR} =\mathrm {\frac{TP}{TP+FN}} \quad \text {and} \quad \text {FPR} =\mathrm {\frac{FP}{FP+TN}} \;, \end{aligned}$$

where TP, FP, TN, FN are defined from the confusion matrix, as shown in Table 3.

To the best of our knowledge, there is no previous research reported on directly using continuous surveillance video sequences for monitoring and early warning of debris flow hazards. Therefore, we compare our method with a binary classifier. The experimental results are performed on the test set of Debrisflow23 (Section 2.1). The ROC curves of the debris flow hazard detection network and debris flow hazard recognition network are shown in Fig. 8. The debris flow hazard detection network achieves 0.863 AUC (Fig. 8a) which outperforms the binary classifier by 36.3 %. We can see the apex of the ROC curve appears at a FPR of 0.3, which is consistent with the analysis of the qualitative experimental results (“Qualitative results”), indicating the network’s accurate and stable debris flow detection capability due to the strongly suppressed false negatives based on the customized loss function (Eq. 5). Figure 8b shows the ROC curve of the debris flow hazard recognition network. It achieves an AUC of 0.837 which outperforms the binary classifier by 33.7 %, making the verification process of potential debris flow hazard false alarms reliable. Note that the performance of the above two models are separately evaluated on the test set, i. e., without the definition of threshold θ in Eq. (6). More importantly, Table 4 presents a comprehensive effectiveness and efficiency evaluation for each component of the proposed system, i. e.,  the video feature extraction network (# 1), debris flow hazard detection network (# 2), debris flow hazard recognition network (# 3), as well as the complete system (# 4) which involves # 1, # 2, and # 3. We can see that # 2 is the lightest model compared to # 1 and # 3, while the 3D feature extraction process of # 1 occupies most of the resource and reduces the efficiency of systematic operation. The overall system (# 4) achieves an AUC of 0.881 with the speed of 68 FPS, outperforming the AUC of # 2 and # 3 by 1.8 % and 4.4 %, respectively. It reveals that the combination of the video-level-based debris flow hazard detection network and the image-level-based debris flow hazard recognition network improves performance effectively and enhances the system robustness.

### Qualitative results

In Fig. 9, we present several qualitative inference results of the proposed system. Intuitively, it can be observed that our method reflects a dynamic anomaly score corresponding to the input video sequence. In particular, anomaly scores are correlated with the magnitude of the corresponding debris flow hazard, where the largest scores occurring at the time of the most severe debris flow movement. At the same time, the dynamic verification process of the debris flow recognition network provides an additional supervision (see the figure titles) to the debris flow hazard detection results, which reduces the false alarm rates. Note that the recognition results of the debris flow recognition network are constantly changing w. r. t. the input video frames. On the contrary, for normal natural videos, the anomaly scores are kept at very low values (nearly zero), suggesting there are no debris flow hazards.

### Failure analyses

The above quantitative and qualitative experimental results and analyses demonstrate the effectiveness of the proposed method. Additionally, we also show two typical failure cases of the debris flow hazard detection network with false alarms (a) and wrong detections (b) in Fig. 10. It can be observed that in the case (a), the yellow dotted box indicates the beginning frame ($53\text {th}$) of a debris flow hazard video, where the debris flow hazard does not occur but the debris flow hazard detection network produces high anomaly scores (false positives). In this case, the introduced debris flow hazard recognition network enables the correct verification of debris flow hazard. In the case (b), the yellow dotted box indicates the ending frame ($602^\text {th}$) of a debris flow hazard video, where the debris flow hazard is still ongoing but the debris flow hazard detection network produces low anomaly scores (false negatives). The reason for these failures is mainly due to the limited availability of debris flow hazard videos, which leads to a lack of data diversity (not all possible combinations of different weather conditions, flow behavior, areas, etc. are presented in the data), which impairs the model performance.

## Discussion

In this study, we introduce a self-assembled Debrisflow23 dataset collected from 7 sampling sites of China and previous recorded debris flow events. The proposed system achieves high accuracy on the current test set. However, the data volume is still relative small compared to popular computer vision tasks, i. e.,  object detection^39,64^, object tracking^65,66^, object segmentation^67–69^, etc. The generalization capability of the system may be degraded due to the complexity and variability of the field environment. Therefore, in our follow-up research, we plan to collect more debris flow hazard videos in larger areas to cover different environmental conditions, thus increasing the data diversity and improving the scalability of the system.

## Conclusion

In this paper, we proposed a unified deep learning-based system for detecting and recognizing debris flow hazards from video streams. It consists of a video feature extraction network, a debris flow hazard detection network, and a debris flow hazard recognition network. These three networks are connected one after the other aiming to provide accurate debris flow hazard identification information. In addition, we introduced the Debrisflow23 dataset, which were collected from 7 sampling sites of China and the previous recorded debris flow events. Extensive experimental results based on the Debrisflow23 dataset show that the proposed system achieves a detection accuracy of 86.3% AUC, a recognition accuracy of 83.7% AUC, and an overall identification accuracy of 88.1% AUC on the test dataset. The promising performance of debris flow hazard detection and recognition indicates the positive impact of the proposed system on mitigating damage and improving early warning capabilities in debris flow-prone areas, which can be implemented for debris flow hazard monitoring and early warning applications.

## Data Availability

The datasets used and analyzed in this study are available from D.L. upon reasonable request.
